# Hospital disaster preparedness in sub-Saharan Africa: a systematic review of English literature

**DOI:** 10.1186/s12873-023-00843-5

**Published:** 2023-06-26

**Authors:** Bashir Farah, Milena Pavlova, Wim Groot

**Affiliations:** 1 Department of Health Services Research, School of Care and Public Health Research Institute, CAPHRI, Maastricht University Medical Center, Faculty of Health, Medicine and Life Sciences, Maastricht University, P.O. Box 616 6200 MD, Maastricht, The Netherlands; 2 Degahbour, Somali Region Ethiopia

**Keywords:** Disaster, Africa, Sub-Saharan Africa, Hospital preparedness

## Abstract

**Background:**

Disasters are increasing worldwide, with Sub-Saharan Africa (SSA) being one of the most prone regions. Hospitals play a key role in disasters. This study provides a systematic review of the evidence on disaster preparedness by hospitals in SSA countries based on English literature.

**Methods:**

A systematic literature review was conducted of articles published between January 2012 and July 2022. We searched PubMed, Elsevier, Science Direct, Google Scholar, the WHO depository library and CDC sites for English language publications. The key inclusion criteria were: publications should have been published in the above period, deal with hospital disaster preparedness in SSA, the full paper should have been available, and studies should have presented a comparison between hospitals and/or a single hospital.

**Results:**

Results indicate improvements in disaster preparedness over time. However, health systems in SSA are generally considered vulnerable, and they find it difficult to adapt to changing health conditions. Inadequately skilled healthcare professionals, underfunding, poor knowledge, the absence of governance and leadership, lack of transparency and bureaucracy are the main preparedness barriers. Some countries are in an infancy stage of their health system development, while others are among the least developed health system in the world. Finally, a major barrier to disaster preparedness in SSA countries is the inability to collaborate in disaster response.

**Conclusions:**

Hospital disaster preparedness is vulnerable in SSA countries. Thus, improvement of hospital disaster preparedness is highly needed.

**Supplementary Information:**

The online version contains supplementary material available at 10.1186/s12873-023-00843-5.

## Introduction

Disasters can lead to severe disruptions of the functioning of a community or society, causing devastation for individuals, as well as economic, material and environmental losses. Disasters may also result in an imbalance between the local supply and demand for health resources while the healthcare infrastructure is frequently damaged by it. Irrespective of whether a health system is well-organized and planned, disasters are a cause for concern. Depending on the magnitude and vulnerability of the populations they affect, disasters frequently exceed the local community’s capacity to respond, needing outside assistance to tackle the event [1–6].

Disasters have increased in frequency and number throughout the world. Globally, the African continent, and sub-Saharan Africa (SSA) in particular, bear the major burden of disasters due to natural forces and man-made events. In the past four decades, disaster has affected over 35 million individuals in SSA, resulting in negative economic and social consequences. Further, nations in Africa are facing a growing assortment of natural hazards, including droughts, volcanic eruptions, floods, and earthquakes, more than other continents in the world [2, 7–14].

The vulnerability of SSA to disasters is caused by a combination of environmental, economic and social factors that negatively reduce the capacity of individuals to secure and protect their livelihoods. In the context of Africa, all countries have nearly similar health system capacity for disaster preparedness. These include a weak and underfunded health system, ill-prepared health facilities, a fragile context, a lack of professional health workforce, equipment, lack of sustainable transportation, prolonged travel time, and poor road infrastructures [15–19].

Due to poor detection and response systems, every year, many SSA countries experience major public health events with a significant risk of a rapid spread. A good example is the Ebola virus epidemic which struck some West African countries in 2014. This public health event has exposed the vulnerability of the population, the economy, and the poor capacities of the affected countries to anticipate and cope with health disasters. Health facilities in many SSA countries lack response plans, surveillance systems, robust preparation and established emergency coordination structures [6, 20–25].

Despite the existence of a Hospital Safety Index (HSI) of the World Health Organization (WHO), which is a checklist of all hazards and a standardized and internationally accepted method for assessing hospital preparedness, there is no legally regulated comprehensive standardized tool to measure hospital preparedness. Hospital preparedness for disasters remains poor and varies substantially within and between SSA countries. Literature suggests that the highest level of hospital disaster preparedness is related to the available human resources. As such, effective preparedness and a comprehensive understanding of disaster management are necessary [26, 27].

Hospitals play a pivotal role in saving lives and reducing the suffering of individuals during and after disasters. Safe and resilient hospitals are structurally strong and organized, offer effective services with a contingency plan, and continue functioning at the utmost capacity during disasters [28, 29]. However, due to the poor level of preparedness, a substantial proportion of deaths in SSA occur in hospital emergency departments. This makes the context and practices of hospital disaster preparedness in SSA countries a critical point of inquiry [3]. However, it remains understudied. Also, little is known about the capacity, capability and preparedness of the SSA hospital infrastructures to manage disasters [4, 6, 28–37].

The aim of this paper is to review the evidence on hospital disaster preparedness in SSA countries based on English literature. Evidence on this topic is important for policy because, in the context of SSA, there is a need to address health system challenges at all levels of healthcare delivery. This paper not only identifies specific gaps in hospital disaster preparedness in SSA countries but also explores the importance of coordinated planning at a regional level to tackle pandemic health threats in SSA countries.

## Methods

The study used the Preferred Reporting Items for Systematic Reviews and Meta-Analysis (PRISMA), also known as the PRISMA statement, to review and report the evidence on hospital disaster preparedness in SSA. The PRISMA 2020 checklist was used to check and confirm that all relevant information was included (see Additional file 1: Appendix A). A review protocol was developed and approved by all researchers. The review protocol was registered with PROSPERO (reference number: CRD42021288155). The final search was done on April 27, 2022, and updated on July 31, 2022.

The review started with a systematic literature search based on predetermined selection criteria and search terms which were developed and approved by all authors. The articles chosen for the review were then evaluated, and their important findings were narratively synthesized based on the review’s objective. The applied review methodology allows for a comprehensive overview of the current knowledge in an exceedingly specific research field.

### Search strategy

We searched PubMed, Elsevier, Science Direct, Google Scholar, the WHO depository library and CDC sites for English language publications from January 1^st^, 2012 to July 31st, 2022. Based on the aim of the review, three groups of search terms were used to build the search terms for the identification of studies on hospital disaster preparedness in SSA nations: (1) disaster; (2) preparedness; (3) Africa or sub-Saharan Africa. Different forms of the above words as well as their relevant synonyms were considered. The keywords utilized to look for relevant literature in the PubMed search engine are shown in Additional file 1: Appendix B.

To maintain uniformity and consistency in the search, MeSH terms (bibliographic thesaurus) were included in the literature search.

The search strategy in Google Scholar, Science Direct or Elsevier and websites of WHO and Centre for Disease Control (CDC) consisted of the same keywords chain. The exact keywords utilized can be found in Additional file 1: Appendix B. The WHO website was searched under the ‘health topics’ to get key health data in SSA. Similar search terms were also used for the search on the CDC website.

### Inclusion and exclusion criteria

To be included in the review, publications on hospital disaster preparedness should have been published between 2012 and 2022 and the full paper should have been available. We purposively selected to include publications not more than 10 years old with the objective of reviewing recent evidence in the study area. The language was limited to English. We included studies that presented a comparison between hospitals and those focusing on one hospital group or a single hospital. Irrespective of their study design, all empirical studies in SSA that met the above criteria were included.

We excluded studies published in non-academic journals, opinion papers, editorials, and review articles available, as well as publications in abstract format. We also excluded studies conducted in healthcare settings other than hospitals and studies from outside SSA, as well as studies that do not directly focus on disaster preparedness and discuss the topic in general. Furthermore, publications that dealt with nosocomial infections or hospital-acquired infections were excluded. In addition, publications for which the full text was not available were excluded.

### Screening process

The initial screening of the publications that appear after searching in PubMed, Elsevier, Science Direct, Google Scholar, the WHO depository library and CDC sites with the set of keywords mentioned above was based on the title and abstract of the publications. During this screening phase, titles and abstracts that have a link to the review topic were considered potentially relevant publications. For the second screening phase, publications were downloaded, and the text of the publication was entirely screened. Publications that met all eligibility criteria outlined above were classified as relevant and were selected for review. The reference lists of the selected publications were also reviewed. In all screening phases, another researcher was involved in diminishing possible selection bias. In each screening phase, the second researcher checked the screening results of the primary researcher. Differences were discussed to reach a consensus on whether to include a given publication.

### Method of analysis

Initially, information on study characteristics was extracted. Then, essential information from each publication was summarized, categorized and clustered. For this purpose, the method of directed qualitative content analysis was applied. As such, the first step is to determine a relevant framework to formulate themes as a guide to the initial code [38, 39]. The theoretical framework for this review was based on the toolkit for assessing health-system capacity for crisis management developed by the WHO to assist countries in reducing the impact of future health crises [3]. According to this WHO toolkit, there are three phases to consider with regard to disaster preparedness, namely the preparation, organization, and reporting phases. For each phase, the following two themes can be defined: (1) context of the hospital or health facility emergency preparedness and (2) practices of the hospital or health facility emergency readiness. The themes facilitated the conceptual understanding of hospital disaster preparedness among health professionals in SSA countries. During the second step, the texts of the selected publications were coded and categorized according to the above themes, which allowed extracting information relevant to this review. Finally, in the third step, the data obtained for each theme were analyzed. The above technique enabled the provision of descriptive knowledge and understanding of the phenomenon under study [39].

The results are shown per theme using a narrative synthesis approach (storytelling) to summarize the relevant findings of this review, with the support of descriptive tables. The application of the narrative approach admits a comprehensive overview of the current knowledge in hospital disaster preparedness.

### Quality check

The validity, reliability and generalizability of the reviewed publications were assessed by the research team in a qualitative manner. We did not use a standardized tool for quality assessment because we included not only empirical papers but also reports. Reliability was assessed by analyzing the sources of the studies, whether the sources were reliable, and whether they were peer-reviewed. Other assessment criteria included questioning the research objectives underlying the study and whether the problem was formulated clearly and logically, and was adequately supported by the literature. If the procedures of data collection and analysis were properly documented in the publication, we classified a study as reliable. Similarly, publications were regarded as valid if the publications showed that the findings were consistent with the study's stated objectives, expectations, and results from previous similar studies. Finally, generalizability was assessed by asking questions related to whether there might be other explanations for the relationships between variables and whether the findings of a study could be transferred to other settings and populations.

## Results

**Fig. 1:**
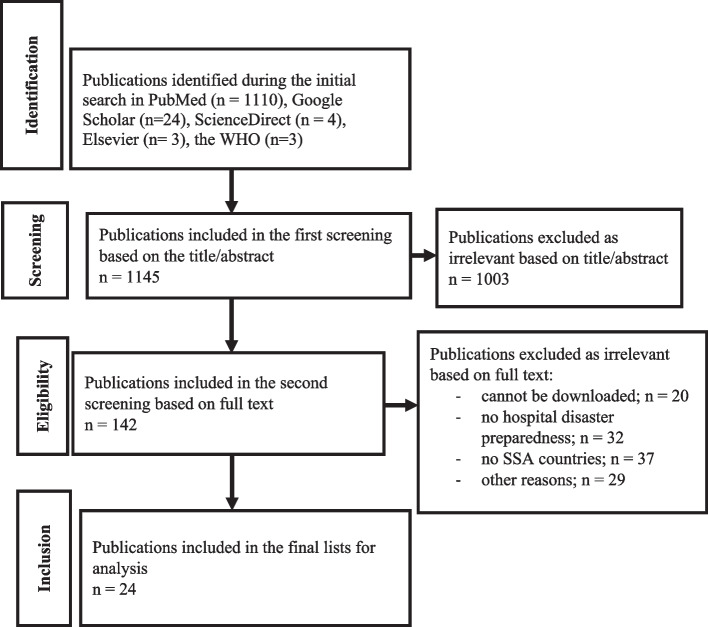
Search results and publication selection procedures

The set of keywords shown above yielded an initial list of 1145 publications, which were included in the initial screening. The results of the screening are presented in Fig. 1. Of the total studies identified, 1003 publications were excluded based on the inclusion/exclusion criteria after reading the title and abstract. In the second screening, 142 publications were included based on full-text availability. For the second screening, the publications were downloaded. The full text of 20 articles could not be obtained, and these articles were excluded. The text of the remaining 122 publications was reviewed entirely. Of these 122 articles, 98 publications were excluded after reading the full text due to the following reasons: publications were not about hospital disaster preparedness, the focus of publications was not on SSA countries, articles were not in English, or there was another reason (e.g., grey literature, books, reports not published in journals or WHO/CDC and documents in other languages). Finally, 24 publications were included in this systematic review. A detailed description of the publications included in this review is shown in Additional file 1: Appendix C.

### General description of selected publications

The overall characteristics of publications included in the review are shown in Table 1. Most of the articles (50%) were published in 2020 and 2022. These recent publications indicate the existence of up-to-date results and the increased attention among academics on the topic. The majority of the articles (70.8%) have been published in the last 6 years. Despite searching for publications in the entire region of SSA, no eligible studies were found for the Central African Republic, Equatorial Guinea, Gabon, Republic of the Congo, and São Tomé and Príncipe. Further, most of the evidence (43% of the reviewed studies) comes from the two East African nations of Ethiopia (33.3%) and Tanzania (16.6%). More than half of the publications examine disaster preparedness in hospitals in SSA countries.

**Table 1:** General description of publications included in the analysis (24 publications reviewed)

Classification category	Subcategories	N (%)	Number in the reference list
Year of publication§	2021–2022	8 (33.33%)	[18, 19, 36, 40–44]
	2019–2020	5 (20.8%)	[4, 17, 20, 45, 46]
	2017–2018	5 (20.8%)	[1, 11–13, 32]
	2015–2016	3 (12.5%)	[2, 16, 31]
	2013–2014	1 (4.16%)	[15]
	2010–2012	3 (12.5%)	[14, 47, 48]
Aim/type of study	Descriptive	20 (83.3%)	[1, 2, 4, 8, 11, 12, 16, 18, 19, 31, 32, 36, 40–43, 45, 47–49]
	Explorative	4 (16.6)	[15, 17, 46, 50]
Research approach	Qualitative (primary data)	12 (50%)	[1, 12, 15–18, 32, 43, 44, 46, 47, 49]
	Quantitative (secondary data)	1 (4.16%)	
	Mixed (primary data)	11 (45.8%)	[2, 4, 11, 19, 20, 31, 36, 42, 45, 48, 50]
Design	Qualitative	12 (50%)	[1, 12, 15–18, 32, 43, 44, 46, 47, 49]
	Quantitative – randomized controlled trial	1 (4.16%)	[40]
	Mixed	11 (45.8%)	[2, 4, 11, 19, 20, 31, 36, 42, 45, 48, 50]
Data collection	Questionnaire	10 (41.6%)	[15–18, 32, 43, 44, 46, 47, 49]
	Key informant interviews	5 (20.8%)	[4, 11, 18, 36, 50]
	Standardized questionnaires/surveys	9 (39.13)	[1, 2, 11, 12, 31, 43, 45, 48, 50]
Reliability is clear	Yes	19 (79.26)	[8, 11, 12, 15, 16, 18, 19, 31, 32, 36, 40–43, 45, 47–49]
	No	5 (20.8)	[13, 14, 17, 41, 46]
Validity is clear	Yes	21 (87.5%)	[1, 2, 4, 8, 11, 12, 15, 16, 18, 19, 31, 32, 36, 40–43, 45, 47–49]
	No	3 (12.5%)	[13, 17, 46]
Generalizability is clear	Yes	21 (87.5%)	[1, 2, 4, 8, 11, 12, 15, 16, 18, 19, 31, 32, 36, 40–43, 45, 47–49]
	No	3 (12.5%)	[13, 17, 46]

The majority of the studies have a descriptive (20 articles) or explorative aim (4 articles) and have a qualitative study design. There are three research approaches in the publications reviewed (qualitative, quantitative and mixed-method approaches). Most of the publications (12 articles) are qualitative studies with primary data collection. The remaining publications are one quantitative with secondary data, and eleven mixed-design studies with primary data.

In Table 1, the assessment of the validity, reliability and generalizability of the studies is presented. If the publications clearly present understandable methods of data collection and analysis, they are considered reliable. In total, 22 publications have clearly described their methods of data collection. The generalizability of 21 publications is outlined, while the validity of some studies is not mentioned.

### Context of hospital or health facility preparedness

The healthcare infrastructure in Africa has longstanding deficiencies. Despite measures taken in terms of the functionality of the health systems, which resulted in improvements in some SSA contexts, SSA health systems are generally considered weak, vulnerable and not adaptable to changing health conditions. The core issues reported in health systems of SSA include inadequately skilled healthcare professionals, underfunding, poor knowledge and lack of training in the use of personal protective equipment (PPE), lack of disaster management policy and plan, the absence of governance and leadership, lack of transparency and bureaucracy, limited capacity, scarce laboratory services, and lack of integration between emergency care to health systems. Such problems are reported in 95% of the publications. Moreover, ineffective healthcare in primary care, the absence of health reporting systems and surveillance, and poor coordination at the national level skewed institutional disaster management systems in SSA countries [15, 16, 40, 45, 47, 51].

Hospitals are an important part of the disaster response system. Yet, most of the reviewed publications indicated that hospitals in the majority of SSA countries had neither a disaster preparedness plan nor other arrangements for the occurrence of disasters. For instance, studies showed a lack of timely access to emergency departments and the use of data that is critical to preparedness in the two most populous nations of SSA, Ethiopia and Nigeria. A further instance of this is an insufficient physical infrastructure which poses a critical challenge to hospitals in SSA countries. Whereas 78% of Tanzanian hospitals are not equipped with basic building services such as dependable electricity and source of water, the existing capacity to provide 24-h emergency care in Rwanda (a country with the highest percentage of hospitals), only 50% of hospitals had the components needed to provide 24-h emergency care. Compared with other SSA countries, Liberia has a fragile health system. At the hospital level, weak supervision, poor quality assurance, inadequate monitoring and evaluation, and lack of job description are reported in Liberia [18, 35, 46].

Addressing issues that hamper hospital disaster preparedness requires close collaboration between all actors in the field. Lack of collaboration between different disciplines in healthcare in SSA countries is a factor that militates against having an optimal level of preparedness. During the Ebola outbreak in 2014, lack of collaboration within the countries and across the West African region has been an issue as well. The collective response to Ebola in West African nations was too little and too late [3, 24, 52].

In Tanzania, only 20% of the regional hospitals had a stockpiling area for the supply of medicines and consumables onsite. Sixty eight percent had a contingency plan that identified a source for these supplies during a disaster. Around 80% had an emergency space for provision of care in surge situations. Only 8.3% of Tanzanian hospitals had temporary mortuaries that were available in the hospitals. In Ethiopia, nearly all hospitals are ill-prepared for potential disaster strikes. Despite gains in South Africa’s health system, the majority of hospitals did not have a dedicated disaster plan training program or had educated their new staff about the institution’s disaster plan [1, 4, 53, 54].

There was not enough hospital disaster preparedness to prevent the COVID-19 pandemic and management in hospitals in North-west Ethiopia. Only one hospital reached an acceptable level of preparedness, even though this hospital had no diagnostic laboratory services for COVID-19. The main challenges that Africa faces in response to COVID-19 pertain to a lack of local biotechnological production and limited research capacity or expertise in specialty fields. This results in African countries being unable to conduct sufficient testing and focused studies on disease transmissibility, vaccine or cure research relevant to the local context [41].

In a study conducted in 25 Tanzanian regional hospitals on the triage, drills and communications during a disaster, around 40% of the hospital areas were designated triage areas for everyday use. There was no validated instrument available for sorting patients, and 60% of the hospitals categorized patients based on the astuteness of an individual provider. Only 32% of the Tanzanian regional hospitals had triage training personnel. Cellular phone communication (96%) was one of the main communication resources relied on during the disaster. The medical officer in charge acts as the main contact person, linking the hospital with other stakeholders in 72% of hospitals [1].

Regarding the context-related barriers to hospital preparedness, one study reviewed key barriers to the provision of emergency in five SSA countries (Ghana, Kenya, Rwanda, Tanzania and Uganda) and found that no surveyed hospital had enough infrastructure to adhere to the minimum standards that the WHO has deemed essential for the provision of emergency care. More importantly, six key areas assessed across all surveyed hospitals, including basic infrastructure, medicine shortage, equipment, infection control, education and quality control, were insufficient and below the internationally acceptable standards. Another study with a focus on climate change and its impacts on human health in six African countries (Ethiopia, Ghana, South Africa, Namibia, Kenya and Nigeria) has also found a serious deficit in all aspects of hospital preparedness in the sample countries to respond effectively to the impacts of climate change. Irrespective of their economic status, across the SSA countries, there are no well-developed hospitals in this region. Accordingly, access to emergency hospital care provided by the public sector in SSA remains poor and varies substantially within and between countries [32, 35, 47, 54].

Based on the findings, we conclude that the overall level of hospital preparedness for emergency and disaster in SSA has shortcomings. Throughout the SSA, there are neither well-developed hospitals nor strong healthcare systems in this part of the world. The malfunctioning of hospitals in SSA was attributed to scarce resources, political instability, poor health leadership, mismanagement, poor coordination between disaster management institutions, and a top-down hierarchical structure which applied in most of the SSA countries [12, 15, 16, 19, 46, 47].

### Practices of hospital disaster preparedness and health professionals in SSA countries

Another problem in hospital disaster preparedness among health professionals in SSA countries is the huge knowledge gap among health professionals. In Ethiopia, the disaster handling preparedness, knowledge and familiarity levels among health cadres are very poor. One of the Southwest Ethiopian studies demonstrated that only 20.6% of the wellbeing health professionals had ever been trained on the preparedness for disaster. Around 92.8% of the health professionals required extra disaster preparedness training and reaction. The majority (71.4%) of health professionals were most interested in the national and local disaster reduction and preparedness plan followed by basic principles and disaster assistance, treatment principles and triage skills, post-disaster psychological relief, post-disaster epidemic prevention, onsite triage and rescue and transport [1, 2, 4, 11, 19, 32, 36, 41].

Knowledge, awareness and experience relating to disaster and disaster preparedness at Tikur Anbessa Specialized Hospital in Ethiopia indicated that 50.8% of the health workers at the hospital had good knowledge about hospital disaster preparedness and its plan, whereas the remaining (49.2%) had low knowledge. Further, very few of them indicated that they knew only the term disaster, while 13.7% of them did not know it. While 26.5% were confused about the term, as they said external help is not needed after a disaster, 4.8% were unsure whether or not external assistance is needed. The main source of information for healthcare workers to increase awareness about disaster and disaster preparedness were books (50%), 16%, 12%, 10% and 7% of respondents get information about disasters from television, radio, other media and newspapers respectively [11].

Another study in Ethiopia reported that 49% of the study participants had good knowledge regarding disaster handling preparedness. In a similar manner, results at Onandjokwe Lutheran Hospital, Northern Namibia, indicated that 42.9% of the participants had fair cognition levels in relation to disaster events in their working environment. The main reason for this positive result might be the participants involved, mainly health professionals working at the emergency department. It is assumed that health professionals working specifically in emergency departments were completely knowledgeable regarding the disaster due to the nature of their working environment as well as their level of training [36].

Woyessa et al. [3] reported that hospital disaster preparedness was weak, with an average calculated preparedness score of 46.6% in the hospitals of Western Ethiopia. This is in line with the findings of Habte et al. [11] for the assessment of knowledge, attitude and practice of disaster preparedness among healthcare workers. Yet, the finding was lower compared to one of the South African studies in Johannesburg Hospital, which assessed hospital disaster preparedness, demonstrated the emergency unit had a preparedness score of 62.5% [55]. A study conducted in three Nigerian states found that none of the hospitals had an emergency operation unit. The indicated differences could be due to the variation in the experience of disaster incidents between Ethiopia and the other SSA countries, resource allocations for hospitals and political commitment. A documented disaster plan, hazard-specific response, and recovery planning were consistently not in place in the evaluated hospitals. This is a terrifying result compared to the findings reported in many African countries, which have at least a documental contingency plan. The managers of the hospitals could face considerable challenges in disaster events, especially logistic deficiency involving a lack of funds, inadequacy of appropriate places to provide medical services, and a shortage of human resources [4, 5, 11, 42, 50, 52, 56].

In Nigeria, the most populous country in Africa with poor disaster preparedness and weak health systems, the issue of hospital preparedness has a lot of challenges. Nigerian hospitals in particular face a major gap between policy commitment and implementation, lack of detailed vulnerability and capacity assessment in setting up healthcare facilities, development and revision of hospital building codes in relation to emerging disasters, lack of continuous training and retraining of healthcare professionals involved in disaster response, lack of evaluation, and absence of considering lessons learned from past disaster experiences [46, 56].

While the level of disaster preparedness in hospitals in South Africa, Namibia and Tanzania is still in its infancy or at an early stage of development. Liberia has the weakest hospital disaster preparedness in SSA countries [18, 19]. This is because Liberia lacks the ability to conduct a pre-planned coordinated response, resources, expertise, accountability and quality control. Prior studies in SSA countries found that there were several gaps in disaster preparedness. Few highly skilled workers tended to be in administrative positions at the hospital, which limited their clinical roles. During the disaster period, the responding personnel might be junior clinical or nursing staff [1, 18, 19, 42, 50, 53, 57].

Another factor is the attitudes and willingness of health cadres to report for duty. The willingness to respond to infectious diseases seems to be related to perceived knowledge. Those who perceived their knowledge to be excellent were generally willing to respond to an infectious disease outbreak, while only 55.6% with good knowledge, 54.8% with fair knowledge, and 51.3% with poor knowledge were willing to respond [50].

In Tanzanian regional hospitals, the human resources available for healthcare delivery were below the recommended ratio for disaster preparedness. Disaster plans and disaster committees are the prime importance for effective management of any disaster as they design a clear plan for how to successfully address disaster-related challenges and outline the roles and required resource allocation during a disaster [58].

In the majority of the publications reviewed, preparedness programs for strengthening emergency and disaster response and recovery in SSA nations were totally absent. Despite a shortage of a multi-cadre professional development strategy in Africa in the past decade, interest in emergency care training is rapidly expanding. However, the absence of training opportunities for health cadres and limited resources pose a critical challenge to SSA’s health sector. Specifically, lack of awareness is the main factor that hampers hospital disaster preparedness. Further, the awareness of disaster plan components is totally missing. Especially, this applies to Ethiopia and Namibia, where essential components of what a disaster plan contains are not clearly known by the practitioners. Apparently, the level of education and training of health professionals in SSA is low. Thus, health professionals require competencies in public health preparedness and response [2, 4, 18, 40, 51].

Merin et al. [59] indicated that triage is a crucial component of routine emergency care and of disaster management. During a disaster event, the mass influx of people in a hospital is likely to add stress to already over-extended hospital staff. During the disaster time, the providers may be pulled from clinical care to attend to their own family members, political leaders, media personnel, and non-critical patients [59, 60].

In recent years, some improvements have been made in the conceptual understanding of healthcare professionals in SSA, mostly as a result of the Ebola outbreak and the involvement of international entities. Across SSA, a considerable number of health professionals had poor knowledge about the concept of disaster preparedness and response to certain specific disasters. In addition to the issues with the low knowledge and limited conceptual understanding of disaster management among health professionals in SSA, issues related to role confusion in disaster incidents, lack of leadership in healthcare, poor governance, lack of integrated information system, weak management, inadequate policy and legal frameworks, and poor accountability has resulted in a gap in the understanding, application and implementation of hazardous event management by health workers in SSA countries [2, 11, 12, 15, 16, 18, 31, 49, 51].

In the studies reviewed, disaster preparedness in SSA countries was generally low at hospital levels. In Ghana, Kenya, Rwanda, Tanzania and Uganda, less than half of all hospitals are able to provide 24-h emergency care, and less than 65% of all hospitals even have basic infrastructure components such as reliable water and power sources. In clinics, the availability of basic infrastructure is even lower, ranging from 7 to 35% of facilities. In Tanzania, regional hospitals had no emergency physicians and no dedicated care nurse training programs. Moreover, only five regional hospitals had a disaster plan and 40% of the hospitals had no disaster committee at all [1, 4, 11, 35, 41, 42, 51].

In Ethiopia, the practices of hospital disaster preparedness are weak [2, 4]. A recent study in selected hospitals in Western Ethiopia demonstrated that the score of readiness in terms of disaster response and recovery planning was 33.3%. Moreover, among the eight hospitals evaluated in North-west Ethiopia, only one hospital reached an acceptable level of preparedness. Besides, disaster medicine was long neglected in Ethiopia as the focus of medical education has been largely clinically oriented. Therefore, the insufficient number of healthcare professionals without adequate training related to disasters and the lack of delivery systems to address practical health problems caused by the impacts of disaster are clear evidence of the ill-prepared situations in the SSA countries to cope and adapt strategies to improve effectively [2, 4, 41, 54].

Barriers related to practices of hospitals, limited surge capacity, low testing ability, weakened health systems, political instability, mishandling of scarce resources, lack of essential services needed during the pandemic (i.e., thermometers, PPE, disinfection detergents, laboratory equipment, equipped rooms, etc.), and the shortage of qualified staff were the major practice related barriers identified across the SSA countries. Furthermore, medical and economic constraints, risk perception apathy among hospital administrators, planning assumptions anticipating orderly and usual events, cost-effectiveness of early preparedness, and business and legal risks have been identified as potential practice-related barriers to hospital disaster preparedness in SSA countries. However, in Nigeria, practitioners' practices regarding the frequency of emergency drills and the frequency of updating emergency plans on a regular basis were grossly inadequate. [1, 6, 16, 19, 31, 40, 42, 43, 49].

## Discussion

This systematic review presents evidence on hospital disaster preparedness in SSA countries. Generally, the healthcare systems of SSA countries are ill-prepared for disasters. Notwithstanding, the evidence obtained through our systematic literature review on this topic indicated a variety of problems related to hospital disaster preparedness. These include weak health systems in which the hospitals operate, inadequate hospital workforce, absence of health leadership in hospital settings, poor governance, hospital underfunding, limited physical access, corruption, system mismanagement, geographical limitations, operational challenges, and lack of knowledge. Furthermore, the lack of equipment and adequate facilities, poor quality assurances, weak supervision, lack of job description, inadequate service integration, and absence of monitoring and evaluation were identified in the review as the key hurdles that hamper the readiness of hospitals for emergency events in SSA.

Our findings have shown that hospital disaster preparedness had shortcomings in the areas like education and training for health workers in most SSA countries, even though in recent years it has been observed increased improvements in awareness, attitudinal change, and conceptual understanding of health professionals. However, the knowledge and training of health cadres are considered a key component of disaster preparedness and prevention of physical damage during a disaster. Similarly, mobilization of additional staff during a disaster, improving training in hospital disaster preparedness and response can also enhance staff performance in other areas of hospital preparedness. Also, a validated triage protocol and training are necessary to ensure effective care and appropriate resource utilization [1, 11].

Studies conducted by Hamer et al. [24] and Chopra et al. [61] indicated that during the Ebola outbreak and COVID-19 pandemics, hospital preparation is the cornerstone for the prevention of the pandemic's spread and reducing the damages. The pandemic preparedness differences depend on the health system and economic level of the country. In a high-income country, for instance, the pandemic preparedness and facility of the health systems were presumably better [24, 61, 62] even though still criticized [63] Kavanagh et al. [62] indicated that many African countries are relying on equipment and reagents imported from outside the continent. The testing capacity was hindered by the inability to rapidly expand the technological capacity, limited funds and, more recently, the export restrictions imposed on COVID-19-related supplies. This result is in line with a study conducted by Barasa et al. [64] which assessed the surge capacity of hospitals, and the result also showed that the hospitals were not well prepared for the COVID-19 pandemic.

We have noted several gaps in disaster preparedness in the Central African Republic, Democratic Republic of Congo, Equatorial Guinea, Gabon, Republic of the Congo, and São Tomé and Príncipe. According to the WHO [65], some of these countries are still recovering from complex emergencies. The current review found that some disasters or outbreaks in Central African countries, including DR Congo and Cameroon, were not reported consistently [66]. However, this does not indicate that up-to-date and evidence on the topic is lacking for a large part of SSA because most of the publications were from East Africa, South Africa and West African countries [65, 66].

SSA countries need to make their health systems more resilient now and in the future. Major factors that prevent strengthening health systems in SSA include inadequate health workforce, absence of strong health leadership and governance, and knowledge gap. On the other hand, Africa faces several other challenges, including armed conflicts, poverty, unemployment, food insecurity, climate change, inequality and industrialization. This complicates the prioritization of health due to competing demands. As a result, health outcomes tend to correlate with donor support. Successful application of improving policy frameworks, training provisions to raise awareness, and improvements on frameworks for information exchange and best practice dissemination across SSA will help close the gap and address the barriers to the successful implementation of adequate coping and adaptation strategies [51, 54, 67].

This indicates that not only is there an inadequate disaster management policy in SSA, but the existence of poor coordination between disaster management institutions at the national level and operational challenges on the ground to respond to an emergency is a critical element for the emergency management cycle. This has distorted the disaster management system in most SSA countries. In order to mitigate morbidity and mortality from a catastrophic surge with limited staff and resources, proposed actions include discharging stable patients from emergency departments and hospitals, canceling elective surgeries, opening alternate care areas, and calling in standby or off-duty staff. However, all these approaches require careful pre-planning [1, 68]. This applies to the Kingdom of Eswatini, a lower-middle-income country of 1.45 million population in southern Africa [15, 43, 64].

The work done by Habte et al. [11] delineated that in disaster planning, the willingness to respond for duty during an occurrence of a disaster is a critical issue. In his study, 95.6% of professionals were willing to report on duty during disaster emergencies. A remarkable number of staff (16.5%) were not interested in reporting duty during disaster incidents. This is in line with a study by Barnett et al. (2009) about attitudes and beliefs toward emergency response among local public health communities. About 16% of the workers were not willing to respond to a pandemic. The common reason mentioned is that “*I am not sure on how I am going to approach a disaster; the hospital might not care for me, and disaster may be frustrating*” [11, 69].

As discussed above, to improve the performance of hospital preparedness for disasters in SSA countries, it is necessary to improve the performance of all disaster-related areas. Education is one of the main pillars for improving disaster management, which should be enhanced through training programs designed for health cadres of SSA nations. The emergency department of a healthcare facility needs planning in the areas of equipment provision, job descriptions, organization of the triage team members, and coherence of the organizational structure of the sector before the occurrence of unexpected hazards. To improve the performance of these categories, recognizing reporting processes, describing communication tasks, using early warning systems, providing equipment, personnel and operational instructions in the area of safety and logistics are other factors that can be used to improve the performance of other areas, which can ultimately lead to the improvement of the overall performance of hospitals in dealing with disasters [3].

Because the challenges faced by the SSA countries in disaster events and their level of development are mostly related, coordinated efforts for effective disaster treatment are required. Given the review results and their discussion, the study recommendations include the need to improve current policies at all levels (i.e., national, regional, and local) on hospital disaster preparedness to adapt to changing health conditions, and to strengthen health professionals’ skills. Trained human power and incorporating disaster management education curricula are highly recommended. Trepidation-related training for those who are afraid to report for duty during infectious disease outbreaks. The regional health offices, universities and other reputable organizations of the countries should support and inspire the hospitals to furnish disaster management education and training to nursing health professionals, particularly for emergency departments. Similarly to this, the emergency care unit professionals are obliged to pay attention to disaster protocols and precede that any catastrophe and emergency events could occur in their working area at any time, and it is crucial that they can make necessary adjustments to their confidence, equipment readiness and cognition. Also, improving the basic knowledge of health institutions to better prepare for all forms of health hazards is recommended. Establishment of coherent and integrated hospital disaster preparedness at national and operational levels is also recommended, next to improving the basic knowledge of health cadres to better prepare and respond to disasters.

This review has some limitations. First, the study has only focused on SSA countries and did not have the ambition to cover the situation of North African countries. This means that results should not be extended to the entire African region. Second, because the study was completely focused on SSA region, issues related to selection bias have been introduced. Future research could consider a comparison between the SSA and North African region. Third, this review was restricted to peer-reviewed research articles and WHO/CDC reports. We did not include grey literature, books, or other documents, which could have also contributed to selection bias. It is possible that there are major incident plans in place that are not published in journals or WHO/CDC reports. Future research could focus on document analysis per country to better understand disaster preparedness in the region. Fourth, we only reviewed English language publications. Thus, publication bias, such as relevant studies not yet published or published in another language, may have occurred. This is particularly relevant for non-English speaking countries in SSA. Fifth, because this study was focused on hospitals in a specific region, SSA, the findings cannot be generalized to other settings. Finally, we cannot completely exclude bias related to the publication selection process, even though we reduced this bias by having two researchers involved in the selection.

## Conclusions

This systematic literature review has analyzed the literature on hospital disaster preparedness focusing on SSA countries. The study concludes that hospitals are the most important institutions in disaster response; they should have prepared and need to have a preparedness plan. The effective means of reducing disaster tragedies by expectancy, alleviating, preparing for, responding to and reclaiming from disaster. Yet, the review indicates that hospitals in SSA are not adequately prepared for possible disaster strikes and emergency situations due to prevailing health system challenges. Key identified challenges are a lack of coordinated multidisciplinary disaster policy and preparedness plan coupled with inadequate health infrastructure, shortage of medical supplies and diagnostic equipment. Also, there is a knowledge and skill gap in emergence triage and disaster response among healthcare providers working from hospitals in SSA countries.

Despite the challenges faced, the interest in emergency care and disaster preparedness is expanding in SSA countries. However, louder actions, including preparing a coordinated strategic multidisciplinary disaster preparedness plan and sound training health system workforce about the effective response to disasters, are needed to introduce real interventions. Thus, increased attention to hospital disaster preparedness is highly needed from Africa’s health system planners’ perspectives and hospital managers to design a sound intervention strategy against disaster in terms of institutional capacities to fully respond to pandemics and other catastrophic disasters involving human causalities.

The SSA health policy-makers and planners should prioritize improving access to healthcare at all levels by investing in healthcare infrastructure at the regional, district and operational levels. This is expected to ensure the availability of medical supplies in hospitals as well as to adopt modern medical diagnostic technologies. The policy-makers should focus on health sector human resource development and building a strong health governance system that can enable coordinated regional-level planning and institutional collaboration to better prepare the health facilities to tackle the pandemic.

Future research can focus on comparative studies on hospital disaster preparedness in private and government hospitals in selected SSA countries, nation-level studies on hospital preparedness in rural and urban areas, and involvement of non-governmental organizations (NGOs) in hospital disaster preparedness in SSA countries, pre- and post-disaster events.

## Supplementary Information

**Additional file 1. **

## Data Availability

PRISMA 2020 Checklist for systematic review and/or meta-analyses is filled in and included in Supplementary file. The list of papers reviewed and keywords used in this review can be found in Supplementary file.

## Abbreviations

CDC: Centre for Disease Control
SSA: Sub-Saharan Africa
HSI: Hospital Safety Index
NGOs: Non-governmental Organizations
PPE: Personal Protective Equipment
PRISMA: Preferred Reporting Items for Systematic Reviews and Meta-Analysis
WHO: World Health Organization
