# Supplementary material for: Hospital disaster preparedness in sub-Saharan Africa: a systematic review of English literature
Source: BMC Emerg Med. 2023 Jun 26;23:71. doi: 10.1186/s12873-023-00843-5 (PMC10291806; doi:10.1186/s12873-023-00843-5)
Supplement: Supplementary file 1 — Additional file 1. [file 12873_2023_843_MOESM1_ESM.docx]

Appendices

Appendix A

The Prisma 2020 checklist

| **Section and Topic** | **Item #** | **Checklist item** | **Location where item is reported** |
| --- | --- | --- | --- |
| **TITLE** | | |  |
| Title | 1 | Identify the report as a systematic review. | 0 |
| **ABSTRACT** | | |  |
| Abstract | 2 | See the PRISMA 2020 for Abstracts checklist. | 1 |
| **INTRODUCTION** | | |  |
| Rationale | 3 | Describe the rationale for the review in the context of existing knowledge. | 2 |
| Objectives | 4 | Provide an explicit statement of the objective(s) or question(s) the review addresses. | 3 |
| **METHODS** | | |  |
| Eligibility criteria | 5 | Specify the inclusion and exclusion criteria for the review and how studies were grouped for the syntheses. | 5 |
| Information sources | 6 | Specify all databases, registers, websites, organisations, reference lists and other sources searched or consulted to identify studies. Specify the date when each source was last searched or consulted. |  |
| Search strategy | 7 | Present the full search strategies for all databases, registers and websites, including any filters and limits used. | 4 |
| Selection process | 8 | Specify the methods used to decide whether a study met the inclusion criteria of the review, including how many reviewers screened each record and each report retrieved, whether they worked independently, and if applicable, details of automation tools used in the process. | 5 |
| Data collection process | 9 | Specify the methods used to collect data from reports, including how many reviewers collected data from each report, whether they worked independently, any processes for obtaining or confirming data from study investigators, and if applicable, details of automation tools used in the process. | 4 |
| Data items | 10a | List and define all outcomes for which data were sought. Specify whether all results that were compatible with each outcome domain in each study were sought (e.g. for all measures, time points, analyses), and if not, the methods used to decide which results to collect. | 4 |
|  | 10b | List and define all other variables for which data were sought (e.g. participant and intervention characteristics, funding sources). Describe any assumptions made about any missing or unclear information. |  |
| Study risk of bias assessment | 11 | Specify the methods used to assess risk of bias in the included studies, including details of the tool(s) used, how many reviewers assessed each study and whether they worked independently, and if applicable, details of automation tools used in the process. |  |
| Effect measures | 12 | Specify for each outcome the effect measure(s) (e.g. risk ratio, mean difference) used in the synthesis or presentation of results. |  |
| Synthesis methods | 13a | Describe the processes used to decide which studies were eligible for each synthesis (e.g. tabulating the study intervention characteristics and comparing against the planned groups for each synthesis (item #5)). | 6 |
|  | 13b | Describe any methods required to prepare the data for presentation or synthesis, such as handling of missing summary statistics, or data conversions. |  |
|  | 13c | Describe any methods used to tabulate or visually display results of individual studies and syntheses. |  |
|  | 13d | Describe any methods used to synthesize results and provide a rationale for the choice(s). If meta-analysis was performed, describe the model(s), method(s) to identify the presence and extent of statistical heterogeneity, and software package(s) used. |  |
|  | 13e | Describe any methods used to explore possible causes of heterogeneity among study results (e.g. subgroup analysis, meta-regression). |  |
|  | 13f | Describe any sensitivity analyses conducted to assess robustness of the synthesized results. |  |
| Reporting bias assessment | 14 | Describe any methods used to assess risk of bias due to missing results in a synthesis (arising from reporting biases). |  |
| Certainty assessment | 15 | Describe any methods used to assess certainty (or confidence) in the body of evidence for an outcome. |  |
| **RESULTS** | | |  |
| Study selection | 16a | Describe the results of the search and selection process, from the number of records identified in the search to the number of studies included in the review, ideally using a flow diagram. | 5 |
|  | 16b | Cite studies that might appear to meet the inclusion criteria, but which were excluded, and explain why they were excluded. | 5 |
| Study characteristics | 17 | Cite each included study and present its characteristics. | 5 |
| Risk of bias in studies | 18 | Present assessments of risk of bias for each included study. |  |
| Results of individual studies | 19 | For all outcomes, present, for each study: (a) summary statistics for each group (where appropriate) and (b) an effect estimate and its precision (e.g. confidence/credible interval), ideally using structured tables or plots. | 7 |
| Results of syntheses | 20a | For each synthesis, briefly summarise the characteristics and risk of bias among contributing studies. |  |
|  | 20b | Present results of all statistical syntheses conducted. If meta-analysis was done, present for each the summary estimate and its precision (e.g. confidence/credible interval) and measures of statistical heterogeneity. If comparing groups, describe the direction of the effect. |  |
|  | 20c | Present results of all investigations of possible causes of heterogeneity among study results. |  |
|  | 20d | Present results of all sensitivity analyses conducted to assess the robustness of the synthesized results. |  |
| Reporting biases | 21 | Present assessments of risk of bias due to missing results (arising from reporting biases) for each synthesis assessed. |  |
| Certainty of evidence | 22 | Present assessments of certainty (or confidence) in the body of evidence for each outcome assessed. |  |
| **DISCUSSION** | | |  |
| Discussion | 23a | Provide a general interpretation of the results in the context of other evidence. | 18 |
|  | 23b | Discuss any limitations of the evidence included in the review. |  |
|  | 23c | Discuss any limitations of the review processes used. |  |
|  | 23d | Discuss implications of the results for practice, policy, and future research. | 22 |
| **OTHER INFORMATION** | | |  |
| Registration and protocol | 24a | Provide registration information for the review, including register name and registration number, or state that the review was not registered. | CRD42021288155 |
|  | 24b | Indicate where the review protocol can be accessed, or state that a protocol was not prepared. | Prospero |
|  | 24c | Describe and explain any amendments to information provided at registration or in the protocol. |  |
| Support | 25 | Describe sources of financial or non-financial support for the review, and the role of the funders or sponsors in the review. |  |
| Competing interests | 26 | Declare any competing interests of review authors. | No |
| Availability of data, code and other materials | 27 | Report which of the following are publicly available and where they can be found: template data collection forms; data extracted from included studies; data used for all analyses; analytic code; any other materials used in the review. |  |

*From:*  Page MJ, McKenzie JE, Bossuyt PM, Boutron I, Hoffmann TC, Mulrow CD, et al. The PRISMA 2020 statement: an updated guideline for reporting systematic reviews. BMJ 2021;372:n71. doi: 10.1136/bmj.n71

For more information, visit: <http://www.prisma-statement.org/>

Appendix B

Keywords per database

**Google Scholar keywords:**

- Group 1 synonyms for disaster: disaster, catastrophe, crisis, public health emergencies, dilemma, setback, problem, misfortune, mass casualty, and mishap.
- Group 2 synonyms for preparedness: hospital preparedness, health facility readiness, hospital response, healthcare system response, emergency management, public health emergency responsiveness, hospital assessment, hospital evaluation, hospital appraisal, planning, checklist, medical services, and medical facility
- Group 3 synonyms for Africa: sub-Saharan Africa, SSA, Africa, developing world, names of the SSA countries.

**Database search in ScienceDirect or Elsevier:**

- Group 1 synonyms for disaster: disaster, catastrophe, crisis, public health emergencies, dilemma, setback, problem, misfortune, mass casualty, and mishap.
- Group 2 synonyms for preparedness: hospital preparedness, health facility readiness, hospital response, healthcare system response, emergency management, public health emergency responsiveness, hospital assessment, hospital evaluation, hospital appraisal, planning, checklist, medical services, and medical facility
- Group 3 synonyms for Africa: sub-Saharan Africa, SSA, Africa, developing world, names of the SSA countries.

**The websites of WHO and CDC keywords chain:**

- Disaster preparedness, public health emergencies.
- Preparedness: hospital preparedness, health facility readiness, hospital response, healthcare system response, emergency management, public health emergency responsiveness, hospital assessment, hospital evaluation, hospital appraisal, planning, checklist, medical services, and medical facility.
- Africa: sub-Saharan Africa, WHO Africa, CDC Africa, SSA, Africa, developing world, names of the SSA countries.

**Database Search in PubMed:**

Group 1: Disaster

**Keywords:**

“Disasters” [MeSH] OR (“disaster” OR “catastrophe” OR “crisis” OR “public health emergency*” OR “dilemma” OR “setback” OR “problem” OR “misfortune” OR “mass casualty*” OR “mishap”)

**MeSH**: Disasters

Group 2: Preparedness

**Keywords:**

“Civil Defense” [MeSH] OR (“preparedness” OR “hospital preparedness” OR “health facility readiness” OR “hospital response” OR “healthcare system response” OR “emergency management” OR “public health emergency response” OR “hospital assessment” OR “hospital evaluation” OR “hospital appraisal” OR “planning” OR “checklist” OR “medical services” OR “medical facility*”)

**MeSH**: Civil Defense

Group 3: Africa

**Keywords:**

“Africa” [MeSH] OR (“Africa” OR “sub-Saharan Africa” OR “SSA” OR “developing world” OR “Horn of Africa” OR “East Africa” OR “West Africa” OR “Central Africa” OR “South Africa” OR “Cameroon” OR “Central Africa Republic” OR “Chad” OR “Congo” OR “Democratic Republic of the Congo” OR “Equatorial Guinea” OR “ Gabon” OR Sao Tome and Principe” OR “Burundi” OR “Djibouti” OR “Eritrea” OR “Ethiopia” OR “Kenya” OR “Rwanda” OR “Somalia” OR “South Sudan” OR “Sudan” OR “Tanzania” OR “Uganda” OR “Angola” OR “Botswana” OR “Eswatini” OR “Lesotho” OR “ Malawi” OR “Mozambique” OR “Namibia” OR “South Africa” OR “Zambia” OR “Zimbabwe” OR “Benin” OR “Burkina Faso” OR “ Cabo Verde” OR “ Ivory Coast” OR “Gambia” OR “Ghana” OR “Guinea” Guinea-Bissau” OR “Liberia” OR “Mali” OR “Mauritania” OR “Niger” OR “Nigeria” OR “Senegal” OR “ Sierra Leone” OR “Togo”)

**MeSH**: “Africa”

***(***“*disaster” OR* “*catastrophe” OR* “*crisis” OR* “*public health emergencies” OR* “*dilemma” OR* “*setback” OR* “*problem” OR* “*misfortune” OR* “*mass casualty” OR* “*mishap”)****AND****(*“*preparedness” OR* “*hospital preparedness” OR* “*health facility readiness” OR* “*hospital response” OR* “*healthcare system response” OR* “*emergency management” OR* “*public health emergency responsiveness” OR* “*hospital assessment” OR* “*hospital evaluation” OR* “*hospital appraisal” OR* “*planning” OR* “*checklist” OR* “*medical services” OR* “*medical facility”)****AND****(*“*Africa” OR* “*sub-Saharan Africa” OR* “*SSA” OR* “*developing world” OR* “*Horn of Africa” OR* “*East Africa” OR* “*West Africa” OR* “*Central Africa” OR* “*South Africa” OR “Cameroon” OR “Central Africa Republic” OR “Chad” OR “Congo” OR “Democratic Republic of the Congo” OR “Equatorial Guinea” OR “ Gabon” OR Sao Tome and Principe” OR “Burundi” OR “Djibouti” OR “Eritrea” OR “Ethiopia” OR “Kenya” OR “Rwanda” OR “Somalia” OR “South Sudan” OR “Sudan” OR “Tanzania” OR “Uganda” OR “Angola” OR “Botswana” OR “Eswatini” OR “Lesotho” OR “ Malawi” OR “Mozambique” OR “Namibia” OR “South Africa” OR “Zambia” OR “Zimbabwe” OR “Benin” OR “Burkina Faso” OR “ Cabo Verde” OR “ Ivory Coast” OR “Gambia” OR Ghana” OR “Guinea” Guinea-Bissau” OR “Liberia” OR “Mali” OR “Mauritania” OR “Niger” OR “Nigeria” OR “Senegal” OR “ Sierra Leone” OR “Togo”)”*

# Appendix C

General description of publications included in the analysis (24 publications reviewed)

| **No** | **Title** | **Authors** | **Year of Publication** | **Characteristics of the studies** | **Characteristics of health-system capacity for disaster preparedness** | | **Barriers to hospital disaster preparedness** | |
| --- | --- | --- | --- | --- | --- | --- | --- | --- |
|  |  |  |  |  | **Context -elated characteristics of health-system capacity for disaster preparedness** | **Practice related capacity for disaster preparedness** | **Context-related barriers** | **Practice-related barriers** |
| 1 | Knowledge, attitude, and practices of emergency health workers toward emergency preparedness and management in two hospitals in Lagos | Adenekan et al., | 2016 | **Aim of Study:**  to assess the knowledge, attitude, and practices of emergency personnel at two tertiary hospitals in Lagos with regard to emergency management and preparedness. **Type of Study:** - Descriptive  **Research Approach:**  Mixed  **Data Collection:**  Self-administered questionnaire  **Country:**  S Africa | ► There was an overall deficiency in the respondents’ knowledge of emergency preparedness.  ► Positive attitude of respondents  toward disaster preparedness. **►** There is a need for improvement in the emergency response so as to match the existing demands of emergency care in the country and worldwide. | ► Most of the respondents did not know when emergency drills  were done in their respective  hospitals | ►Lack of knowledge on disaster  preparedness | ► The frequency of emergency drills and the frequency of regularly updating the emergency plans were grossly inadequate |
| 1 | Level of emergency and disaster preparedness of public hospitals in Northwest Ethiopia: A cross-sectional study | Ayenew et al., | 2022 | **Aim of Study:**  To determine the level of hospital emergency and disaster preparedness at public hospitals in the east Gojjam zone of Northwest Ethiopia.  **Type of Study:** - Descriptive  **Research Approach:**  Mixed  **Data Collection:**  World Health Organization observation checklist **Country:**  Ethiopia | ►The evaluated hospitals’ overall level of emergency and disaster preparedness falls into the low category, with an average calculated preparedness score of 54.75 %  ►In most hospitals, there is a plan in place to ensure the continuity of essential clinical support, as well as a potential triage aria and referral system for emergencies. However, there is no plan in place to provide  psychosocial support to victims of an  emergency | ►Finance and logistics were the lowest prepared sectors for disasters and emergencies.  **►** Patient care and support services were the highest prepared services for emergencies (60 %). | ►There is no system in place to assess the personnel performance.  **►** There is no plan for ensuring security or evacuating victims when an emergency occurs | **►** Human resources are critical in the care of victims of disasters or health emergencies.  ► There is no system in place to assess the personnel performance. |
| 3 | General overview of the disaster management framework in Cameroon | Bang, | 2014 | **Aim of study:**  **-**to review disaster management in Cameroon, exam- ining the various legislative, institutional, and administrative frameworks that help to facilitate the process.  **Type of Study:**  **-**qualitative  **Research approach:**  **-**primary  **Data collection: -**empiricaldata **Country:**  **-**Cameroon | ► Despite constructive measures, Cameroon has failed to achieve reasonable success in disaster management. | ► Inadequate disaster management policies, poor coordination between disaster management institutions at the national level, the lack of trained disaster managers, a skewed disaster management system, and a top-down hierarchical structure within Cameroon’s disaster management framework. | ► The disaster management process generally pursues a top-down hierarchical approach to the administration and  implementation of activities, with more emphasis on disaster response than on risk prevention and mitigation.  ► Although government policy on civil protection in the country recognizes other state and nonstate actors, their role in disaster management is not very clear.  ► There is a gap in understanding, applying and implementing policies, with potentially dire ramifications for the management of hazardous events.  ► There are no comprehensive and explicit laws that show how the disaster management policy is applicable to institutions and to the managerial process for the entire territory. There are no comprehensive strategies and | ► The economic, environmental, physical, and social factors that negatively affect the capacity of people to secure and protect their livelihoods. |
| 4 | Knowledge, Experiences and Training Needs of Health  Professionals about Disaster  Preparedness and Response in Southwest Ethiopia: a cross sectional study | Berhanu et al,. | 2016 | **Aim of Study:**  to assess the perceived knowledge, experiences and training needs of health professionals regarding disasters, their prevention and management in Jimma Zone, Southwest Ethiopia. **Type of Study:** - Descriptive  **Research Approach:**  -Mixed  **Data Collection:**  **S**tructured questionnaire **Country:**  Ethiopia | ► Ethiopia's health system is ill-prepared for disasters. | ►Although the majority had good knowledge about the public health consequences of  disaster in general, a considerable number of health professionals had limited knowledge about the concept of disaster and response to certain specific disasters. | ► Limited resources | ► Huge knowledge gap exist among the health professionals. Apparently, education and training of health professionals is low.  ► Health professionals require competencies in public health preparedness and response.  ► Disaster medicine was long neglected in Ethiopia as the focus of medical education has been largely clinically oriented. |
| 5 | African National Public Health  Institutes Responses to COVID-  19: Innovations, Systems  Changes, and Challenges | Binder et al., | 2021 | **Aim of study:**  **- t**o provide ideas to help NPHIs as they continue to address the COVID-19 pan- demic and prepare to respond to future public health emergencies.  **Type of Study:**  **-**descriptive  **Research approach:**  **-**quativelita **Data collection: -**systematic **Country:**  **-**SSA (Africa) | ► Public health infrastructure in Africa has a longstanding deficiencies and preparedness is not fully ensured. | ► Health systems have been overwhelmed by the COVID-19 pandemic. | ► Lack of new systems of data  sharing, technological innovations, new approaches to public–private partnerships, and strengthening of public health capacity in areas distal to the central government have been needed. | ► Increased visibility, expansion of partnerships, workforce development, commodities issues, genomic capacity gaps, addressing non-Covid-19 priorities and addressing inequities |
| 6 | Assessment of Knowledge,  Attitude and Practice of  Disaster Preparedness among  Tikur Anbessa Specialized  Hospital Health Care Workers,  Addis Ababa, Ethiopia | Habte et al., | 2018 | **Aim of Study:**  to assess current awareness, attitude, and practice of the health care workers regarding disaster preparedness and to find out what arrangements were in place should a disaster occur at the hospital.  **Type of Study:** - Descriptive  **Research Approach:**  Mixed  **Data Collection:**  self-administered questionnaires and semistructured key **informant interview Country:**  Ethiopia | ► Health system is not well prepared for disasters and the hospital is poorly prepared for disasters. | ► Most health care  practitioners lacked the  knowledge and management  skills to deal with disasters.  ► Moreover, the hospital had neither disaster preparedness plan nor other forms of arrangements and preparations for occurrence of disasters. | ► Lack of Awareness is the main factor that hampers disaster preparedness. The awareness of disaster plan components is missing. This applies to Ethiopia and Namibia where essential components or what disaster plan contains is not clearly known by the practitioners.  ► Developing culture of preparedness remain a challenge to national authorities. | ► Inadequate knowledge on role of hospital could lead to role confusion during disaster incidents.  ► Practitioners lack knowledge and capability to respond for disasters. |
| 8 | Using critical information to strengthen pandemic preparedness: the role of national public health agencies | Khan et al., | 2020 | **Aim of Study:**  to strengthen national accountability and preparedness for infectious diseases. **Type of Study:** - Explorative  **Research Approach:**  Qualitative  **Data Collection:**  Key informant interviews (NPHIs) **Country:**  Ethiopia and Nigeria | ► Public health systems and capacities are  insufficiently prepared to prevent a localized infectious disease outbreak from spreading. | ► Lack of timely access to, and use of, data that is critical to preparedness. | ► Long delays in accessing data on priority indicators to detect or respond to an outbreak.  ► Lack of mechanisms for receiving data from private healthcare providers. | ► NPHIs need to urgently be equipped with the mandate, expertise and resources to carry out a broader role in preparedness.  ► NPHIs must have the authority to request quick sharing of data from public and private sector organizations during health emergencies and to access additional human and financial resources during disease outbreaks. |
| 9 | Fire disaster preparedness and situational analysis in higher learning institutions of Tanzania | Kihila | 2017 | **Aim of Study:**  **-** To investigate the level of fire disaster preparedness considering the availability and condition of firefighting facilities as well as the knowledge on fire management among the selected 10 higher learning institutions.  **Type of Study:**  -Descriptive  **Research Approach:**  **-Qualitative Data Collection:**  -Structured questionnaire **Country:**  **-**Tanzania | ► Institutions are not well prepared to manage fire or disaster outbreaks | ► Facilities were not regularly serviced; not enough water storage, respondents had never received any training on firefighting and prevention; institutions are not well prepared to manage fire outbreaks. lack of a natural disaster management plan for the country. | ► Facilities are not regularly serviced  ► Stored hazard materials  ► Lack of enough water storage for firefighting and fire assembly point | ► Lack of knowledge and training  ► Institutions are not well-prepared for disasters |
| 10 | Disaster preparedness and response capacity of regional hospitals in Tanzania: a descriptive cross-sectional study | Koka et al., | 2018 | **Aim of Study:**  to assess the current state of disaster preparedness and response capacity among Tanzanian regional hospitals. **Type of Study:** - descriptive  **Research Approach:**  Qualitative  **Data Collection:**  Prospective (structured questionnaire)  **Country:**  Tanzania | ► Hospital disaster preparedness is at an early stage of development in Tanzania.  ► Lack of disaster planning in more than half of the regional hospitals.  ► Only five (20%) had a disaster plan.  ► Forty percent of the hospitals had no disaster committee at all. | ► All the hospitals were found to have inadequate numbers of all cadres of health care providers to support effective disaster response.  ► No hospital had all components of surge capacity.  ► Only five (20%) of the hospitals had a stockpiling area with supplies (medications and consumables onsite).  ► Potential gap in emergency preparedness and response capability of hospital staff. | ► Human resources available for health care delivery at each regional hospital are below the recommended ratio for all the cadres. | ► Lack of additional staff to mobilize during a disaster. |
| 11 | Readiness and early response to COVID-19: achievements, challenges and lessons learnt in Ethiopia. | Lanyero et al., | 2021 | **Aim of Study:**  To discuss the challenges and lessons learnt which could be used to inform future management of health emergencies.  **Type of Study: -** Descriptive  **Research Approach:**  **-Qualitative Data Collection: - Survey Country:**  Ethiopia | ► Ethiopia demonstrated adequate readiness capacities in coordination structures, case management, infection  prevention and control (IPC), logistics, risk  communication and community engagement and surveillance. | ► Limited capacities were noted in laboratory capacity and rapid response teams.  ► Operational readiness to respond to an emergency is a critical element of the emergency management cycle. | Key challenges for readiness.  ► First, multisectoral coordination was weak. The existing multisectoral platform known as the emergency coordination committee chaired by the NDRMC and bringing together various government sectors and partners in emergency preparedness and response was not fully functional over a long period. ► The huge influx of over 10000 returnees from different COVID-19-affected countries over a short period of time constrained response resources. For instance, additional quarantine and isolation facil- ities had to be established with provision of essential health services, mental health and psychosocial support and rehabilitation services. | ► The multiple health emergencies in the country, such as yellow fever, cholera, dengue, measles, disease outbreaks, flooding and population displacement, further strained the health system.  The existing shortage of health workers, medical supplies.  ► The shortage of PPEs in the country resulting from the global supply chain disruption of the importation. Lack of PPE stockpiles in country and limited capacity for local production increased the risk of healthcare worker infections. |
| 12 | Ebola impact on African health systems entails a quest for more international and local resilience: the case of  African Portuguese speaking countries | Lapão et al., | 2015 | **Aim of Study:**  **-**To identify the most important Ebola outbreak impact factors on the African health systems.  **Type of Study:**  **-**Descriptive  **Research Approach:**  **-Qualitative and Quantitative Data Collection:**  **-**literature review and case study **Country:**  **-**African Portuguese speaking countries | ► Health facilities are ill-prepared and vulnerable, with few resources and equipment. | ► Healthcare services cannot give a good clinical or nourishment answer.  ► It is very difficult for the healthcare systems to respond without weakening the response of other services, often left without capacity.  ► Health professionals are easily affected by epidemics. This disrupts the recruitment of new health professionals.  ► Careless to use biosafety equipment or personal protectives.  ► Lack of training in the use of PPE  ► The lack of rigor in the technical approach has caused a growing distrust and alarmism in populations.  ► Pressure to diagnose epidemic disease, which often leads to | ► Limited resources and conflict | ► The shortage of qualified staff, lack of equipment and adequate facilities and equipment (i.e., thermometers, PPE, disinfection detergents, laboratory equipment, equipped rooms, etc.) |
| 13 | Building a new platform to support public health emergency response in Africa: the AFENET Corps of Disease Detectives, 2018–2019 | Masiira et al., | 2020 | **Aim of Study:**  **-**To review public health emergency preparedness in SSA **Type of Study: -**explorative  **Research Approach:**  **-**Qualitative **Data Collection: - Survey Country:**  **-**Uganda and Zimbabwe | ► Lack of skilled health workforce and finance | ► Inadequate skilled public health workforce and underfunding. | ► Lack of effective community engagement can result into violence against the rapid responders. | ► Deficiencies in financing the health sector in SSA is feasible. |
| 14 | Assessing the preparedness of primary healthcare facilities during a cholera outbreak in Kinshasa, Democratic Republic of the Congo, 2018 | Ndumbi et al., | 2020 | **Aim of Study:**  to assess the level of preparedness of HCs in responding to cholera outbreaks  **Type of Study:** - Descriptive  **Research Approach:**  Mixed  **Data Collection:**  A structured questionnaire**Country:**  Democratic Republic of the Congo | ► Low level of cholera preparedness.  ►The overall level of preparedness of frontline  HCs was alarmingly poor in the city of  Kinshasa  ►Only 5% of preparedness were met  ► poor health service provision in the country | ► The existence of a fragile health system  **►** Necessary investments are not made to meet the defined preparedness | ► Unimproved water source inside the health system facility.  **►**Gaps in basic life-saving supplies that are essential **for** case management, such as oral rehydration salts (ORS) (35%) and Ringer lactate (24%). The lack of these supplies could lead to an exacerbation of case fatality during a cholera outbreak. | ► Serious gaps in WaSH conditions with only  ► 9.4% reporting a functional hand washing station inside the latrine ► Insufficient quantity of chlorine. |
| 15 | COVID-19: lessons and experiences from South Africa’s first surge | Moonasar et al., | 2021 | **Aim of Study:**  **-**To describe measures taken by South Africa to contain the spread of COVID-19 and to mitigate its effects. **Type of Study: -Descriptive**  **Research Approach:**  **-**Qualitative  **Data Collection:** Survey **Country:**  **-**South Africa | ► The South African healthcare system is  two tiered, comprising of a public, government-run sector and a private sector | ► The services in the public healthcare sector are divided into primary (clinics), secondary (district and regional hospitals) and tertiary (academic) health services.  ► The National Department of Health is responsible for the development of health-related policies and guidelines, which are then implemented at provincial and district levels. | ► System mismanagement including corruption | ► Challenges included the delayed approval of the surge strategy and the absence of a single integrated information system at hospital level that could be used to track demand and guide the supply of the appropriate healthcare services; confusion surrounding the multiple different forecasting models in circulation and supplier’s inability to meet the sudden increase in demand.  ► Limited supplies of personal protective equipment (PPE) |
| 16 | Hospital disaster emergency preparedness: A study of Onandjokwe Lutheran Hospital, Northern Namibia | Ncube & Chimenya | 2016 | **Aim of Study:**  To explore disaster emergency preparedness at Onandjokwe Lutheran Hospital in Northern Namibia. **Type of Study:** - Explorative  **Research Approach:**  -Mixed  **Data Collection:**  self-administered questionnaire, semi-structured key informant interviews, and a hospital disaster plan checklist.  **Country:**  Namibia | ► Preparedness process is still in its infancy stage. | ► A shortage of skilled human resources is a major impediment in most African countries.  ► Lack of trained staff in emergency preparedness and response in many countries in the region | ► Developing a culture of preparedness remains a challenge for most national authorities.  e | ► Attitudes and willingness to report for duty. The willingness to respond to infectious disease seems to be related to perceived knowledge. All of those who perceived their knowledge to be excellent were willing to respond to an infectious disease outbreak, while only 55.6% with good knowledge, 54.8 % with fair knowledge, and 51.3% with poor knowledge were willing to respond. |
| 17 | Hospital all-risk emergency preparedness in Ghana | Norman et al., | 2012 | **Aim of Study:**  the paper assessed the emergency prepar- edness programs of health facilities for all-risks but focused on Road Traffic Accidents, (RTA) resulting in surge demand  \**Type of Study:** - Descriptive  **Research Approach:**  Mixed  **Data Collection:**   - site visit and questionnaire survey,**Country:**   GHANA | ►Many of the nation’s hospitals were not prepared and did not possess general  emergency pre- paredness programs  ► There is no clear plan  **►** The existence of emergency planning group | ► Inadequacies of the hospital system in responding to emergencies raise serious public health concerns. **►** The triage standards designed by the Ghana Health Service, (GHS) and distributed to all Government Hospitals is flawed  because it did not pro- vide for field or on-site care management as reported. | ►Lack of pre- emergency and emergency  preparedness plans as well as the coordination of the hospitals response mecha- nisms. | ► Lack of competent medical and health personnel **►** Lack of adequate supplies. |
| 18 | Access to emergency hospital care provided by the public sector in sub-Saharan Africa in 2015: a geocoded inventory and spatial analysis | Ouma et al., | 2018 | **Aim of Study:**  to complete a geocoded inventory of hospital services in Africa in relation to how populations might access these services in 2015, with focus on women of child bearing age. **Type of Study:** - Descriptive  **Research Approach:**  -Qualitative **Data Collection:** -Survey **Country:**  SSA | ► Physical access to emergency hospital care provided by the public sector in Africa remains poor and varies substantially within and between countries. | ► Emergency care which refers to the health system capacity required to ensure effective provision of curative services for life-threatening events is not given attention in SSA countries.  ► Integration of emergency care to health system is required. | ► High population density and high hospital density. ► Geographical accessibility to emergency hospital care varied between countries  ► Only 16 SSA countries met the international recommendation of more than 80% of the population within 2-h travel time of a hospital. | ► Long distance travel for the nearest hospital.  ► The hospital had neither disaster preparedness plan nor other forms of arrangements and preparations for occurrence of disasters. |
| 19 | Emergency care capacity in Africa: A clinical and educational initiative in Tanzania | Reynolds et al., | 2012 | **Aim of Study:**  To describe an initiative at Muhimbili National Hospital in Dar es Salaam, with a focus on the development of the emergency medicine residency program.  **Type of Study:** - Descriptive  **Research Approach:**  -Qualitative **Data Collection:**  Survey  **Country:**  Tanzania | ► Health systems in sub-Saharan Africa are weak and vulnerable. There is no welldeveloped facilities in this part of the world. | ► There were no emergency physicians in Tanzania and no dedicated emergency care nurse training programs | ► Despite shortage of a multi-cadre professional development startegy in Africa, interest in emergency care training is rapidly expanding in SSA. | ► The burden of acute disease in sub-Saharan Africa is severely under-documented.  ► Health-care facilities often lack an integrated approach to triage, resuscitation, and stabilization of acutely ill patients.  ► Essential components of acute and emergency care have not been determined, and there is no consensus on how to define success.  ► There is no current advocacy plan for placing emergency care o the global health agenda |
| 20 | Overview of preparedness and response to COVID-19 in Ghana | Sarkodie et al., | 2021 | **Aim of Study:**  -To provide an overview of preparedness and response to COVID-19 pandemic in Ghana over the period January to December 2020. **Type of Study:**  -Exploratibe  **Research Approach:**  -Qualitative **Data Collection: -**Survey **Country:**  **-**Ghana | ► Good governance and leadership are crucial for effective, efficient, and successful management of pandemics and other complex public health emergencies, and Ghana’s experience has demonstrated this. | ► Health sector driven preparedness included infrastructure, equipment, logistics and human resource capacity development and enhancement, public education and sensitization, public and stakeholder engagements.  ► Key activities for preparedness included the training of healthcare workers on the disease based on existing knowledge. Training also focused on surveillance using developed case definition on COVID-19, Infection Prevention and Control for diseases spread by droplet in- fection and the appropriate use of Personal Protective Equipment (PPE). | ► Geographical limitations and operational challenges, like distribution of food had resulted dissatisfactions | ► Sporadic short ages of PPEs, lack of staff to manage treatment centres and shortage of COVID-19 laboratory reagents. |
| 21 | Strengthening the community health program in Liberia: Lessons learned from a health system approach to inform program design and better prepare for future shocks | Simen-Kapeu et al., | 2021 | **Aim of Study:**  to improve the health status of the Liberian pop- ulation through building a resilient health system that contributes to achieving equitable health outcomes  **Type of Study:**  -Descriptive  **Research Approach:**  Qualitative  **Data Collection:**  Key informant inter- views and focus group discussions **Country:**  Liberia | ► Fragile health system | ► Weak coordination and leadership.  ► Inefficient distribution  ► Inadequate inventory management | ► Poor governance  ► Weak Management  ► Inadequate policy and legal framework  ► Limited funding  ► Poor accountability | ► Lack of job description  ► Lack of coordination on training  ► Inconsistent incentives  ► Poor geographic accessibility  ► Inadequate service integration  ► Weak supervision  ► Poor quality assurances  ► Inadequate monitoring and evaluation |
| 22 | Emergency and Disaster Handling Preparedness Among  Front Line Health Service Providing Nurses and  Associated Factors at Emergency Department, at  Amhara Regional State Referral Hospitals, Ethiopia | Tilahun et al., | 2021 | **Aim of Study:**  To identify knowledge of frontline health-care nurses with regard to accident and disaster preparedness handling at Amahara Regional State Referral Hospitals.  To determine attitudes of emergency department nurses to accident and disaster preparedness handling.  To distinguish level of familiarity of emergency department nurses with accident and disaster prepa- redness handling. - To explore related factors of frontline working nurses’ accident and disaster preparedness handling.  **Type of Study:** -Descriptive  **Research Approach:**  **-mixed**  **Data Collection:**  -Key informant interview **Country:**  Ethiopia | ►Ill-prepared for disasters | ► Poor knowledge of disaster handling preparedness. | ► Disaster handling preparedness, knowledge and familiarity levels were below | ► Inadequate knowledge in relation to disaster readiness |
| 23 | A cross-sectional survey of COVID-19 preparedness in governmental hospitals of North-West Ethiopia | Tiruneh et al., | 2021 | **Aim of Study:**  to assess the level of hospital preparedness for COVID-19 in South Gondar Zone Governmental Hospitals, 2020. **Type of Study:** - Descriptive  **Research Approach:**   - Qualitative   **Data Collection:**  Institution based survey**Country:**  Ethiopia | ► Hospital disaster preparedness in Ethiopia is very poor.  ► Hospitals were in an acceptable level of preparation.  ► There was no hospital prepared enough  ► Pandemic preparedness in Ethiopia is 52% and community needs awareness, sanitation measure, and fulfillment of the materials required for the pandemic. | ► Drugs, personal protective equipment (PPE), and laboratory services were scarce and need immediate action before the overwhelming of the pandemic | ► Insufficient infrastraucture. There were no laboratory services to diagnose COVID-19 immediately. | ► There was no laboratory diagnostic method and treatment center for the COVID-19 virus.  ► Lack of components like putting the patient in an isolated and wellventilated room, availability of adequate PPE, avoidance of patient movement from room to room, and practice in recording the entry of persons to the patient room.  ► Limited human resource capacity. insufficient levels of PPE, including masks, gloves, and hand sanitizers, and insufficient water supply from all hospitals. |
| 24 | Disaster Preparedness in Selected Hospitals of Western Ethiopia and Risk Perceptions of Their Authorities | Woyessa et al., | 2020 | **Aim of Study:**  to assess disaster preparedness level in selected hospitals of the western part of Ethiopia and their authorities’ risk perceptions.  **Type of Study:** - Descriptive  **Research Approach:**  Mixed  **Data Collection:**  Key informant interview**Country:**  Ethiopia | ► The overall level of emergency and disaster preparedness in the selected hospitals was weak. ► The score of readiness in terms of disaster response and recovery planning was 33.3%. | ► Hospitals were ill-prepared for the potential disaster strikes. No committee was responsible for emergency readiness. | ► The preparedness programs for strengthening emergency and disaster response and recoverywere totally absent. | ► An emergency operation Centre (EOC) that must have been available in a safe, secure, and accessible location with immediate opera- tional capacity were also not designated. |
